# Electrochemical Proton Storage: From Fundamental Understanding to Materials to Devices

**DOI:** 10.1007/s40820-022-00864-y

**Published:** 2022-06-14

**Authors:** Tiezhu Xu, Di Wang, Zhiwei Li, Ziyang Chen, Jinhui Zhang, Tingsong Hu, Xiaogang Zhang, Laifa Shen

**Affiliations:** Jiangsu Key Laboratory of Electrochemical Energy Storage Technologies, College of Material Science and Technology, Nanjing University of Aeronautics and Astronautics, Nanjing, 211106 People’s Republic of China

**Keywords:** Electrochemical proton storage, Rapid kinetics, Charge storage mechanism, Material design, Device construction

## Abstract

Simultaneously improving the energy density and power density of electrochemical energy storage systems is the ultimate goal of electrochemical energy storage technology. An effective strategy to achieve this goal is to take advantage of the high capacity and rapid kinetics of electrochemical proton storage to break through the power limit of batteries and the energy limit of capacitors. This article aims to review the research progress on the physicochemical properties, electrochemical performance, and reaction mechanisms of electrode materials for electrochemical proton storage. According to the different charge storage mechanisms, the surface redox, intercalation, and conversion materials are classified and introduced in detail, where the influence of crystal water and other nanostructures on the migration kinetics of protons is clarified. Several reported advanced full cell devices are summarized to promote the commercialization of electrochemical proton storage. Finally, this review provides a framework for research directions of charge storage mechanism, basic principles of material structure design, construction strategies of full cell device, and goals of practical application for electrochemical proton storage.

## Highlights

- Fundamental principles and advantages of electrochemical proton storage are briefly reviewed.
- Research progresses and strategies to promote the development of electrochemical proton storage based on various charge storage mechanisms, electrode materials, and devices are discussed and summarized.
- Challenges and perspectives of the next-generation electrochemical proton storage technology are discussed.

## Graphical abstract

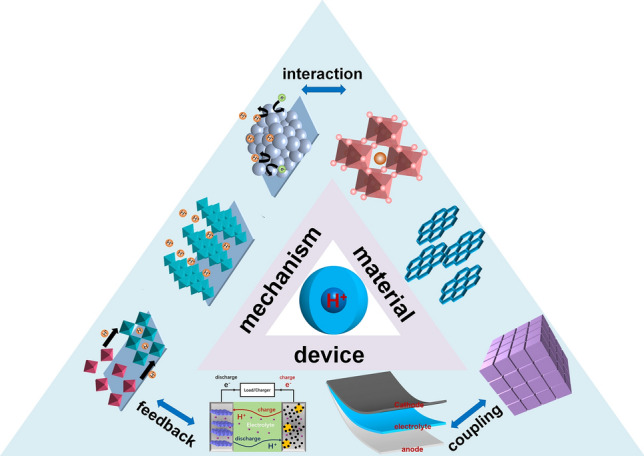

## Introduction

With the increase in demand for energy conversion and storage owing to the development of green energy technology, grid-scale energy storage is playing a more and more important role in the development of energy [1]. Electrochemical energy storage systems provide an effective strategy for improving the reliability and utilization of power grids [2]. Lithium-ion batteries (LIBs), as the best among them, have been widely studied all over the world. Recently, some promising metal batteries with lithium anodes have been the focus of research for higher energy density, such as Li–S batteries [3, 4], Li–O_2_ batteries [5, 6], and Li–CO_2_ batteries [7, 8]. However, the limitation of lithium resources and the strong demand for higher energy density and higher power density push the development of next-generation rechargeable batteries to replace current LIBs [9]. Therefore, a series of other metal ion charge carrier batteries have been developed, such as sodium-ion batteries (SIBs) [10], potassium-ion batteries (PIBs) [11], zinc-ion batteries (ZIBs) [12], magnesium-ion batteries (MIBs) [13], and aluminum-ion batteries (AIBs) [14]. Despite most of them deliver a high energy density, the power density of metal ion charge carrier batteries is very low due to the slow solid-state diffusion process during the electrochemical reaction. Furthermore, safety is also a common problem in metal ion charge carrier batteries, which limits their further development in some key areas. In order to meet the diverse demand for energy, various aqueous non-metal carrier batteries and supercapacitors with excellent electrochemical performance have become a hot topic in energy research [15–19].

Proton, the ion with the smallest molar mass, is an ideal charge carrier. Such a small ion mass will effectively decrease the mass burden of the electrode material, thereby leading to a higher capacity of the electrode [20]. For example, for a one-electron reaction in MoO_3_, a hypothetical HMoO_3_ electrode delivers a theoretical specific capacity of 185 mAh g^−1^, which is higher than the theoretical specific capacity of LiMoO_3_ (177 mAh g^−1^) and NaMoO_3_ (160 mAh g^−1^). In addition, proton also has the smallest ionic radius (∼10^−15^ m) and smaller hydrated ionic size (2.82 Å for H_3_O^+^), which contributes to the rapid migration of protons in electrolytes and electrodes. Typically, H_2_SO_4_ is one of the electrolytes with the highest ionic conductivity among all electrolytes. As early as 1802, Grotthuss proposed the Grotthuss conduction similar to Newton’s cradle to explain the curious ion conductivity of acidic aqueous solutions [21]. Specifically, one water molecule combines with a proton and throws away another proton from the other end of the water molecule; then, the released proton is transferred to the next water molecule through a hydrogen-bonding chain, triggering a series of reorganizations of the hydrogen-bonding network and leading to the rapid transport of protons [22, 23]. Likewise, studies have shown that protons are transferred through hydrogen-bonding networks in hydrous oxides and MOFs following the Grotthuss mechanism [24, 25]. Different from obvious structural change in electrode material derived from intercalation of large-sized metal carriers during the charging and discharging process, the electrode structure distortion caused by protons with small ion radius can be ignored during the cycle. In summary, the superiority of proton carriers will endow electrochemical proton storage (EPS) with higher energy, fast chargeability, long cycle life, and other excellent electrochemical performance. In recent years, many proton batteries and pseudocapacitors with higher capacity, ultra-higher rate, and unparalleled cycle life have been reported [21, 26–30].

Unfortunately, although a lot of research has been done to develop EPS systems with excellent electrochemical performance in recent years, the huge gap between practical problems and ideal designs still restricts the practical application of EPS. At present, the development of electrode materials and the research of electrochemical reaction mechanisms for EPS are in their infancy, and it is still challenging to develop proton pseudocapacitors/batteries with fast charging capability and high stability. This article comprehensively reviews the development history of electrochemical proton storage materials and summarizes advanced electrochemical proton storage materials. These materials possess unique physicochemical properties compared with traditional electrode materials, which requires further understanding of the relationship between microstructure and electrochemical performance. This review also emphasizes the fundamental mechanism of electrochemical proton storage from atomic-scale electrochemistry, such as the law of charge transfer, the characteristic of proton transport, and the interaction mechanism between electrode materials and charge carriers. In order to develop high-performance devices, some advanced pseudocapacitors and batteries are discussed. From the perspective of experimental research and practical application, the roadmaps for the mechanism research, the electrode design principles, and the commercialized application of the next-generation EPS are proposed.

## Protons as Charge Carriers

### Advantages of Electrochemical Proton Storage

**Fig. 1:**
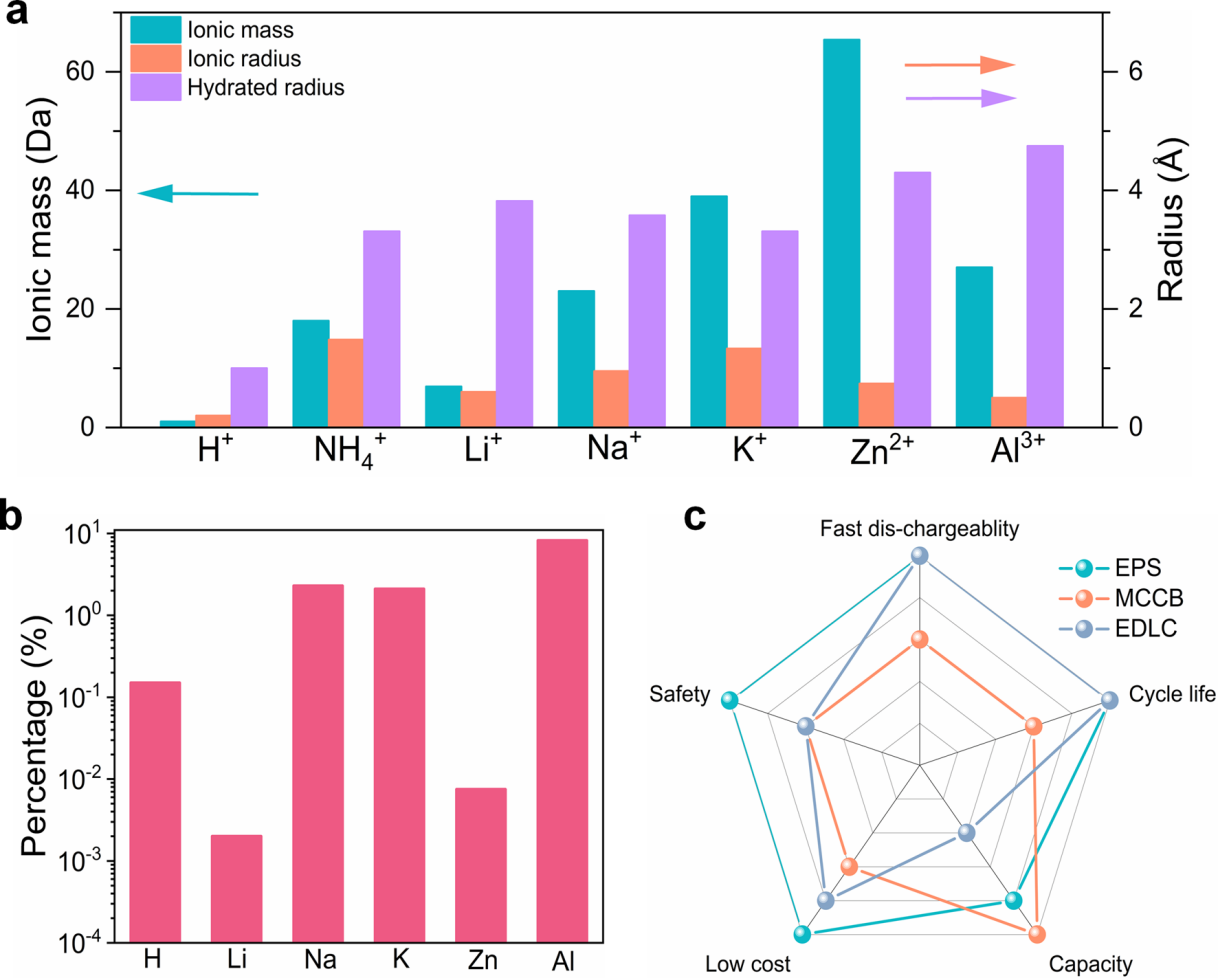
**a** Ionic mass, ionic radius, and hydrated radius for typical cation charge carriers. **b** The abundance of various elements in the crust. **c** Radar plot comparing the property of different electrochemical energy storage systems, where EPS represents the electrochemical proton storage, MCCB represents metal ion charge carrier batteries, and EDLC represents electric double-layer capacitors

To surpass the power limitations of metal ion charge carrier batteries and capacity limitations of electrical double-layer capacitors, more attention has been devoted to the research of non-metal ion charge carrier batteries. An electrochemical energy storage system that uses protons (H^+^) as charge carriers is gradually appearing in people's vision, which is attributed to the following advantages: (i) excellent electrochemical performance, the proton has the smallest ionic radius and the smallest atomic mass, which can endow electrodes with high energy density, fast kinetic reactions, and long cycle life when comparing with traditional charge carriers (Fig. 1a, c) [31]. (ii) Low cost, hydrogen is almost the most abundant element on earth (the percentage of hydrogen element is dozens of times higher than that of lithium element only in the Earth's crust), which directly reduces the cost (Fig. 1b). Moreover, the cost derived from the process of material preparation and equipment operation is greatly decreased, because proton batteries/pseudocapacitors mainly operate under a hydrous and oxygenic environment [32]. (iii) Environmental benignity, electrochemical proton storage systems frequently use acidic aqueous solutions as electrolytes to avoid the environmental pollution caused by the toxic organic electrolytes. (iv) Safety, active metals (such as Li, Na, and K) and flammable organic solvents are not used in EPS to reduce the occurrence of safety accidents such as spontaneous combustion. Specifically, proton batteries have been demonstrated to possess extremely outstanding electrochemical performance at especially low temperatures [33–40], which will be useful in military, polar, aerospace, and other fields.

### Electrochemical Proton Storage Mechanism

**Fig. 2:**
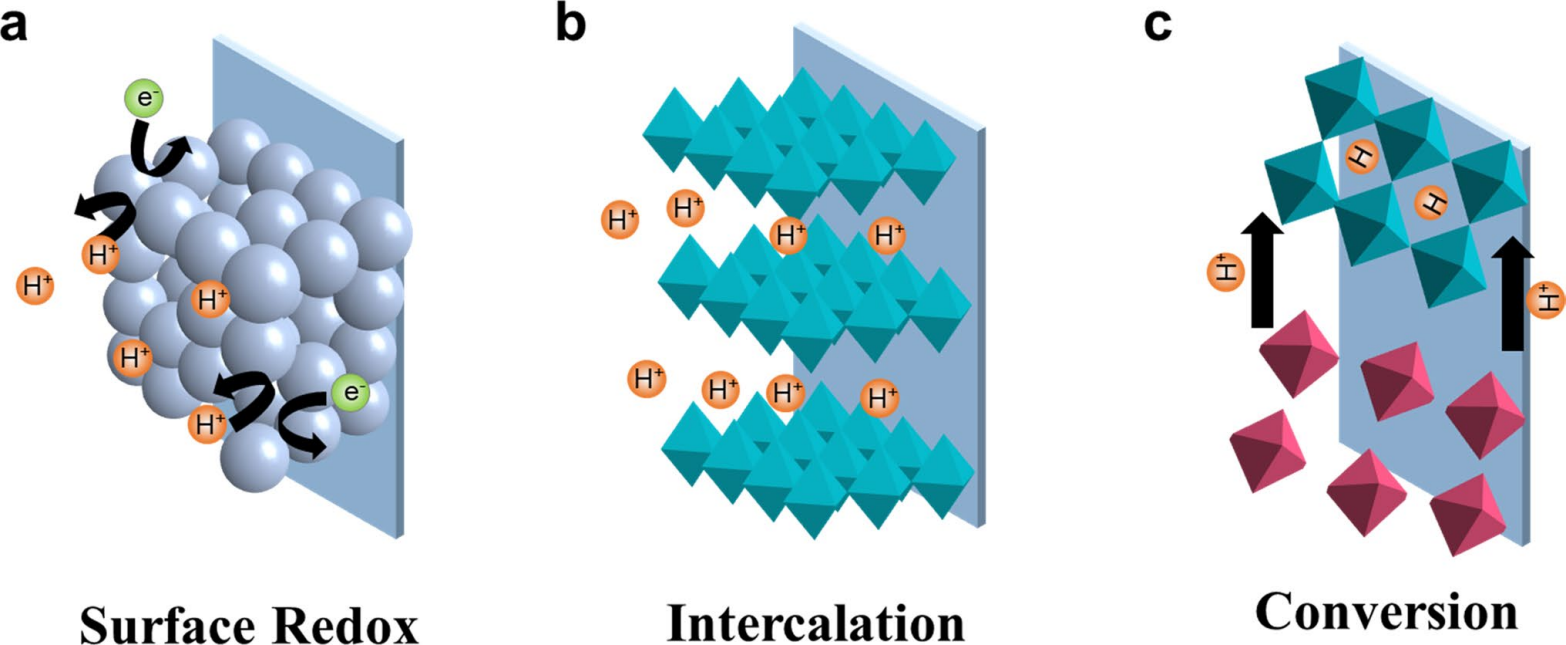
Illustration of three types of charge storage mechanisms for electrochemical proton storage. **a** Surface redox reaction mechanism. **b** Intercalation reaction mechanism. **c** Conversion reaction mechanism

EPS is classified into three types based on their energy storage mechanisms: surface redox reaction mechanism, intercalation reaction mechanism, conversion reaction mechanism. (i) During the surface redox reaction, charge storage occurs on or near the electrode surface, leading to ideal capacitive behavior [41]. Protons will be electrochemically adsorbed, and a rapid kinetic reaction is completed in abrupt diffusion owing to the short diffusion distance (Fig. 2a). This surface redox reaction mechanism often occurs in RuO_2_. (ii) For the intercalation reaction mechanism, protons are inserted into the electrode bulk phase and the electrode structure remains stable during the charging/discharging process, which ensures the electrode good cycle stability (Fig. 2b). In particular, intercalation reaction of metal ion charge carriers often progresses very slowly and is accompanied by a phase change (the main reason is that metal ions form ionic bonds with O or other anions in the electrode bulk phase). In contrast, the intercalation of protons is generally very rapid and undergoes a non-phase change (this is caused by the reason that protons form covalent-ionic bonds with other atoms in the electrode bulk phase) [31]. According to the recent research, electrochemical proton storage materials based on intercalation reaction mechanisms are divided into the following categories: metal oxides (such as WO_3_), two-dimensional transition metal carbides/nitrides (MXenes), and Prussian blue analogs (PBAs). (iii) Regarding the conversion reaction mechanism, the electrode materials will undergo structural conversion and chemical bond reorganization that is often accompanied by phase changes during the charging and discharging process, thereby releasing a huge capacity (Fig. 2c) [42]. Therefore, by breaking the limitation of the structure, the capacity of the conversion electrode material can be several times that of the intercalation-type material. Conversion electrode materials mainly include metal oxides (MoO_3_) and organic materials (quinones, conductive polymers, covalent organic framework, etc.).

## Electrochemical Proton Storage Materials and Mechanisms

Electrode materials are important compositions for electrochemical proton storage, ideal electrode materials should have the following characteristics: (i) large capacity and wide potential window, (ii) fast proton transport kinetics, (iii) long cycle life, (iv) low cost and pollution. Currently, electrode materials for electrochemical proton storage have been initially developed, it is necessary to summarize and classify reported electrode materials to provide more valuable information for readers and researchers. Based on different charge storage mechanisms, electrode materials can be classified into surface redox reaction materials (RuO_2_), intercalation reaction materials (WO_3_, PBAs, and MXenes), and conversion reaction materials (MoO_3_ and some organic materials) according to the recent research. In this section, the basic properties of electrode materials and the interaction mechanism between electrode materials and protons are mainly discussed.

### Surface Redox Reaction Materials

RuO_2_ is a classic material based on the surface redox reaction mechanism. In 1995, Zheng et al. reported for the first time that the electrochemical performance of RuO_2_ depended on its crystal structure [43]. The amorphous phase-hydrated RuO_2_ possessed a specific capacitance of 720 F g^−1^, while the specific capacitance of highly crystalline RuO_2_·nH_2_O was only about one-tenth of the former. Additionally, they also found that protons intercalated into the bulk of hydrous RuO_2_ more easily than RuO_2_ with the crystalline phase due to the presence of water molecules in hydrous RuO_2_. Dmowski et al. further revealed the influence of RuO_2_·nH_2_O structure on physical and chemical properties by X-ray diffraction (XRD) and atomic pair density function (PDF) [44]. They found that the capacitance of RuO_2_·nH_2_O was closely related to structural water content (when *n* = 0.58, RuO_2_·0.58H_2_O achieved a maximum capacitance of 850 F g^−1^), and the changes of water content directly affected electronic and protonic penetration pathways. Furthermore, the PDF results indicated that the metallicity of hydrated RuO_2_ came from the anhydrous rutile-like nanocrystals of RuO_2_ and crystal water provided an efficient transport network for protons. Such nanostructure and crystal water endowed hydrated ruthenium oxide with not only high proton conductivity but also high electronic conductivity, pushing that RuO_2_·nH_2_O was widely used in the energy storage and conversion field. More importantly, this also provided important ideas for the design of proton–electron mixed conductors and proton capacitive materials.

**Fig. 3:**
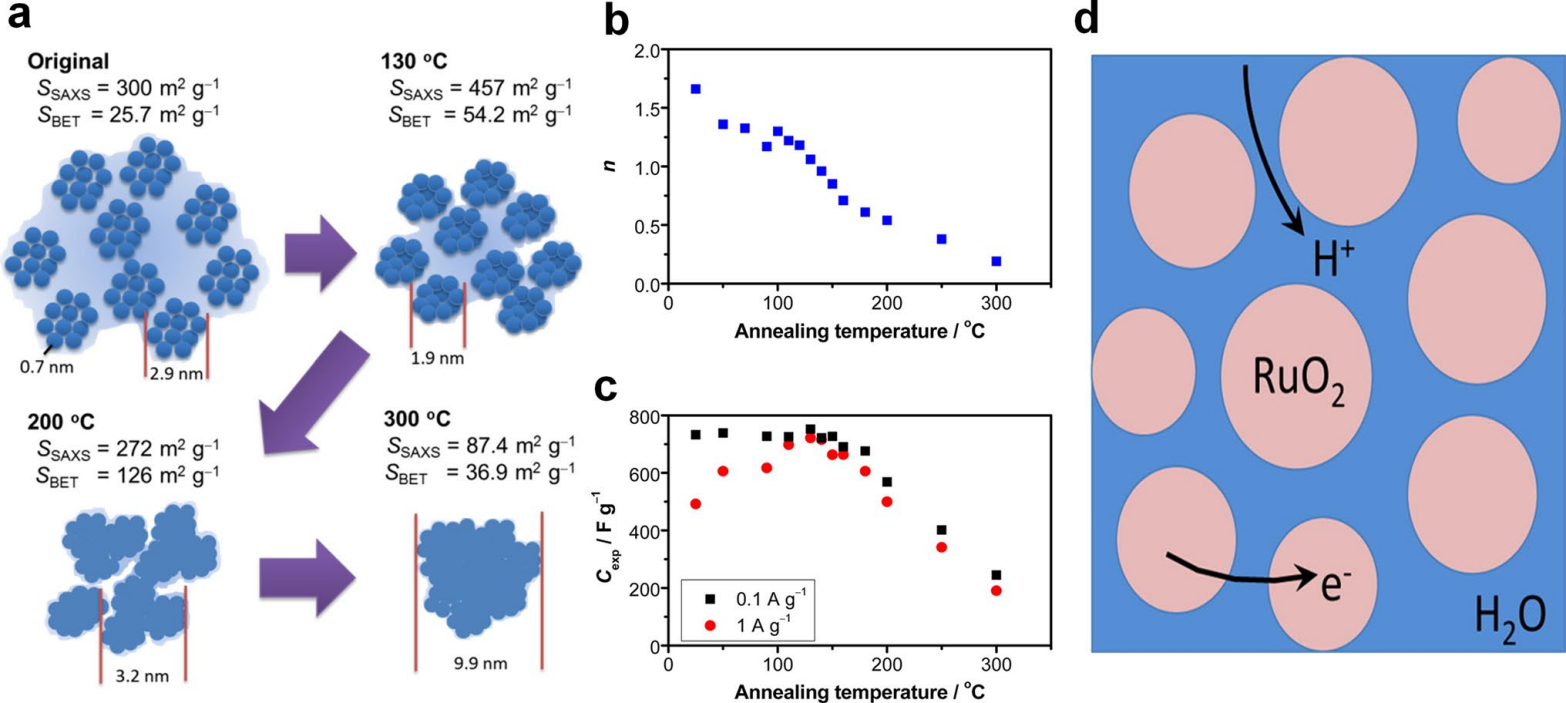
**a** The relationship between specific surface area and particle size of RuO_2_·nH_2_O. **b** Water molecule content of RuO_2_·nH_2_O at different annealing temperatures. **c** Specific capacitance of RuO_2_·nH_2_O at different annealing temperatures [45]. Copyright 2013, American Chemical Society. **d** Schematic of proton charge transfer mechanism on RuO_2_ surface. Ruthenium, oxygen, and hydrogen ions are shown as blue, red, and pink circles, respectively [46]. Copyright 2013, American Chemical Society

Yoshida et al. used small-angle X-ray diffraction (SAXA) to quantitatively study the relationship between RuO_2_ sub-nanostructure and surface area and successfully revealed the origin of pseudocapacitance [45]. Specifically, the agglomeration state and specific surface area of RuO_2_ particles were different at different annealing temperatures (Fig. 3a), and the specific capacitance increased with the increase of the specific surface area of the material (the specific capacitance was 720 F g^−1^ at 457 m^2^ g^−1^, while the specific capacitance was less than 600 F g^−1^ at 272 m^2^ g^−1^) (Fig. 3b–c). The above results proved that the pseudocapacitance of RuO_2_ came from the surface redox reaction, not the bulk reaction of RuO_2_. In order to better understand the charge storage mechanism of RuO_2_ in acidic media, more and more researchers have begun to use theoretical calculation methods to analyze the thermodynamic and kinetic behavior of RuO_2_ on the atomic scale [46–49]. The results of first-principles density-functional theory (DFT) showed that the intercalation of protons would lead to the formation of O–H covalent bonds and the formation of an electronic delocalization state for only 0.3 electrons transferring to the Ru atom center. When the voltage was lower than 0.1 V, the energy barrier of proton migration reached 1.8 eV, which demonstrated that the kinetics of protons were controlled by diffusion. When the voltage shifted to a higher voltage, additional protons tended to be adsorbed on the active sites of the RuO_2_ surface to form functional groups such as hydroxyl groups (−OH). Overall, these conclusions support that unusual pseudocapacitance of RuO_2_ comes from the charge transfer of protons on the grain boundary formed by water molecules and RuO_2_ (Fig. 3d).

### Intercalation Reaction Materials

#### ***WO***_***3***_

**Fig. 4:**
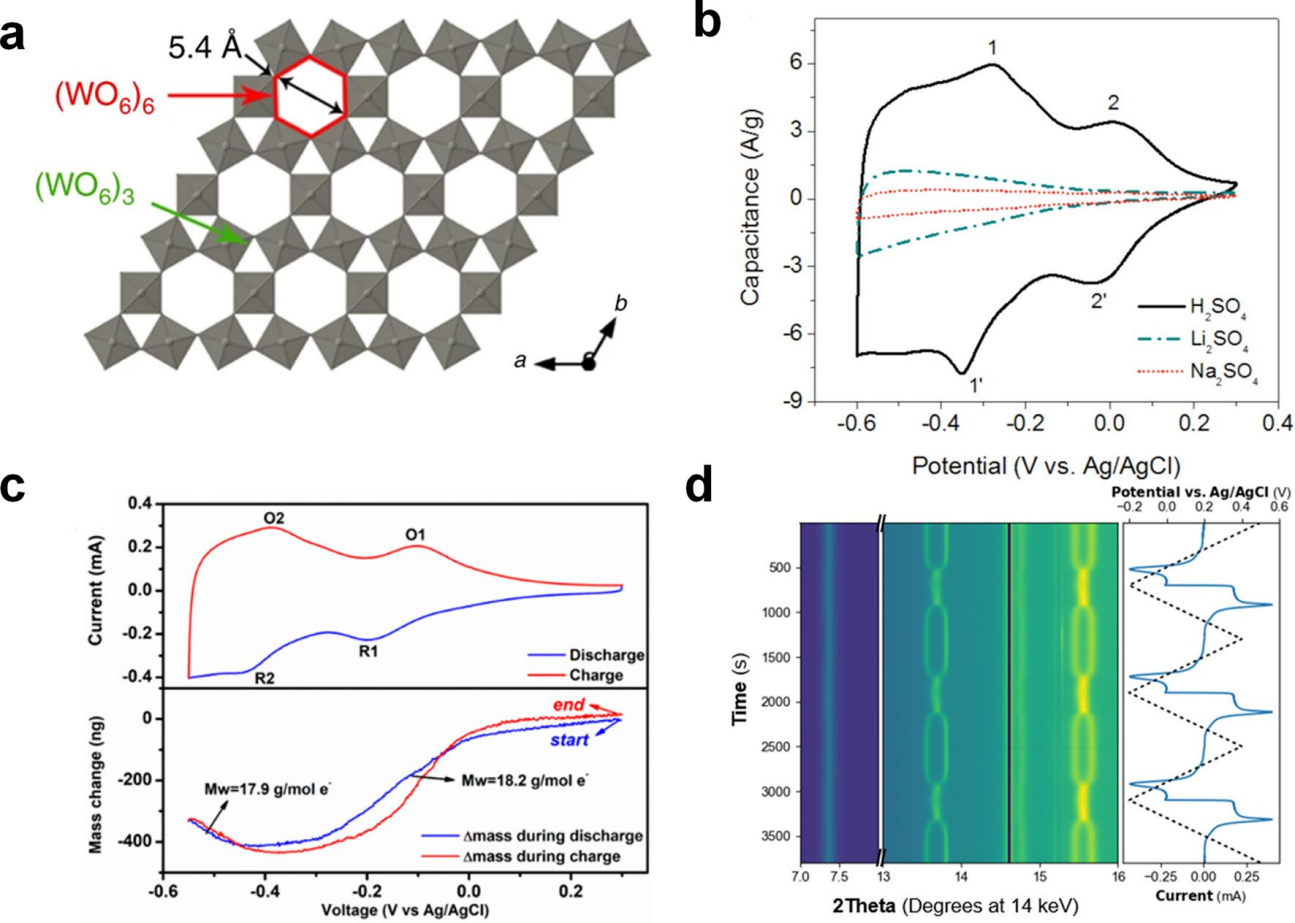
**a** Schematic diagram of the electronic structure of $h$-WO_3_ [59]. Copyright 2019, Springer Nature. **b** CV curves of *h*-WO_3_·0.6H_2_O in different electrolytes [26]. Copyright 2015, American Chemical Society. **c** The CV curves (top) and corresponding EQCM curves (bottom) of *h*-WO_3_·0.6H_2_O at 3 mV s^−1^ [63]. Copyright 2018, American Chemical Society. **d** In situ XRD study of electrochemical phase transition of WO_3_·2H_2_O at 1 mV s^−1^ [67]. Copyright 2019, American Chemical Society

WO_3_ is a typical oxide that crystallizes with the ReO_3_-type structure (only ReO_3_ and WO_3_ in binary oxides) [50], which includes a WO_6_ octahedral network with shared angles in three dimensions and a W–O–W bond angle of between 165° and 179° [51]. Additionally, compared with other perovskite-type structures, the more open structure of ReO_3_-type tungsten oxide is conducive to the intercalation and extraction of various charge carriers (Fig. 4a) [52]. WO_3_ was initially used in the research of electrochromism where WO_3_ film exhibited better color-changing ability, resulting from the co-intercalation of protons and electrons in H_2_SO_4_ solution [53–56], which made it possible for WO_3_ to be used in electrochemical proton storage. Similar to RuO_2_·nH_2_O, crystal waters play an important role in the superfast transport of protons and the increase of specific capacity [57]. Mitchell et al. found that the presence of crystal water seriously affected the electrode reaction kinetics, and the electrochemistry reaction changed from battery to pseudocapacitive behavior with the change of crystal water content [58]. Particularly, WO_3_·2H_2_O electrode delivered a specific areal capacitance of 0.25 F cm^−2^ at a high loading mass of 4.57 mg cm^−2^ (charging or discharging only took 5 s). Chen et al. reported a hydrated $h$-WO_3_ with high proton conductivity (*σ*_H_∼1 mS cm^−1^) and electronic conductivity (*σ*_e_ ∼ 0.6 S cm^−1^), possessing excellent electrochemical performance [26]. On the one hand, high specific capacitance (498 F g^−1^) of $h$-WO_3_ was derived from high specific surface area and open channels [59, 60]. On the other hand, the rapid migration of protons and electrons led to ultrahigh rate performance (over 80% capacitance retention at 100 mV s^−1^). Additionally, the capacitance was almost not attenuated after 50,000 cycles because of the negligible strain caused by protons with small volumes in the electrode. Due to the excellent physical and chemical properties of WO_3_ materials, the application of WO_3_-based EPS in photoelectric integration and thermoelectric integration will be the direction of vigorous development in the future [61, 62].

However, the charge storage mechanism of tungsten oxide in acidic media is still controversial [59, 63–66]. The first crucial question is whether ions inserted into the electrode material are only protons (maybe H_3_O^+^ or even water molecules). Jiang et al. used a quartz crystal microbalance (QCM) to probe the relationship between mass changes and charge changes during the charge and discharge of $h$-WO_3_·0.6H_2_O [63]. They found that the reaction process was not a simply proton intercalation process, but involved multi-step reactions and the intercalation of a variety of ions (Fig. 4c). For a protonation process: first, WO_3_·0.6H_2_O absorbed ∼0.29 H_2_O when it came into contact with the electrolyte; secondly, the electrode excluded ∼0.25 H_2_O and acquired ∼0.25 H^+^; then, ∼0.3 H^+^ was inserted into the electrode; finally, an intercalation process of ∼0.17 H_3_O^+^ was found. Besides, another important question is what kind of role lattice water plays in the electrochemical reaction process. Many studies assumed that protons could be transported through the Grotthuss mechanism in materials with structural water, indicating that protons can jump very quickly on the hydrogen bond network. Wang et al. used operando atomic force microscopy dilatometry to detect the degree of deformation of WO_3_ and WO_3_·2H_2_O during the redox process [66]. They found that the deformation of WO_3_·2H_2_O was lower than anhydrous WO_3_ at a scan rate of 20 or 200 mV s^−1^. (This corresponded to the better kinetics behavior of WO_3_·2H_2_O.) Furthermore, by analyzing the local structural deformation rate combined with ex situ XRD, they revealed that the distortion of WO_3_ was three-dimensional, while the distortion of WO_3_·2H_2_O was two-dimensional because of the presence of structural water. In order to understand the function of structural water in proton transport and faraday reaction, Mitchell et al. used operando XRD technology and solid-state nuclear magnetic resonance (SSNMR) to monitor the electrochemistry process of proton intercalation/de-intercalation in WO_3_·nH_2_O and WO_3_ [67]. The two pairs of (200)/(001) and (101)/(011) diffraction peaks of hydrated WO_3_ were highly reversible during charging and discharging at a scan rate of 1 and 100 mV s^−1^, demonstrating that WO_3_·2H_2_O had a reversible phase transition during the de/intercalation of protons (Fig. 4d). On the contrary, the structure of WO_3_ could not respond quickly to electrochemical protons intercalation because there was no obvious structural transformation of WO_3_ at 100 mV s^−1^. Thus, the above conclusions not only proved that the structural water ensured the stability of electrode structure and accelerated the reaction kinetics of electrode, but also provided a novel strategy for the development of high-power energy storage devices via taking advantage of the flexibility of confined fluids.

#### Prussian Blue Analogues (PBAs)

**Fig. 5:**
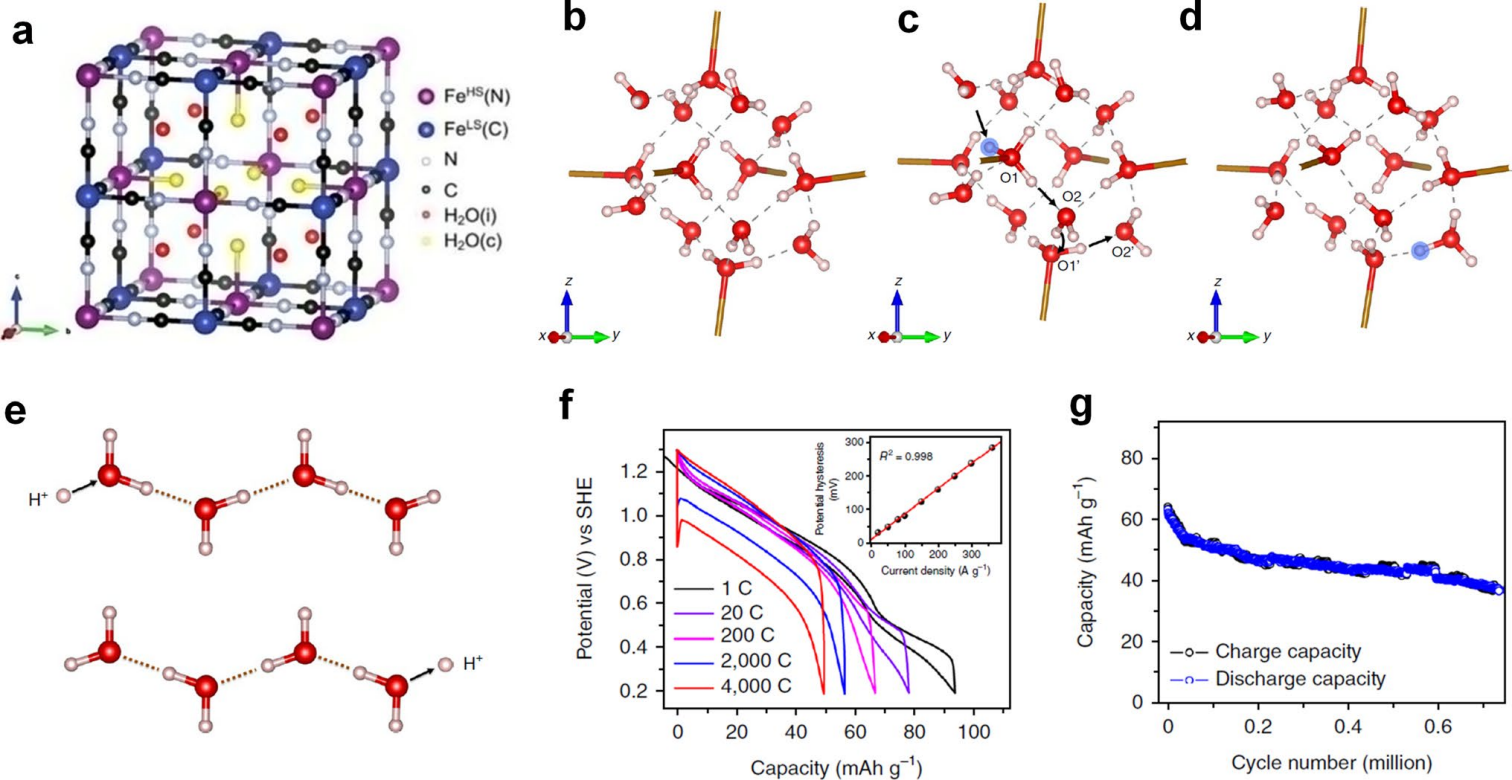
**a** Schematic of crystal structure of Fe_4_[Fe(CN)_6_]_3_·14H_2_O [68]. Copyright 2020, Wiley–VCH. **b**–**d** The transport path of protons in CuFe-TBA. **e** Schematic of Grotthuss mechanism. **f** GCD curves of CuFe-TBA at different current rates. **g** Cycle performance of CuFe-TBA at a current density of 500 C [21]. Copyright 2019, Springer Nature

Prussian blue analogues (PBAs) are a class of substances with a molecular formula A_x_M_1_[M_2_(CN)_6_]._y_□_1-y_·zH_2_O, where A represents alkali metal element (such as K, Na, and Li), M is transition metal element (M_1_ may be Fe, Mn, Zn, etc., while M_2_ usually is Fe, Co or Mn), □ represents vacancy, and water molecules are mainly divided into free water, coordination water, and zeolite water (Fig. 5a) [68, 69]. These ions and molecules are alternately connected to each other to form a cubic open frame. Additionally, rigid crystal lattices and open-dimensional tunnels facilitate charge carriers diffusion in PBAs, leading to widespread use of PBAs in the field of electrochemical energy storage [21, 38, 70–75]. However, PBAs prepared by the liquid deposition method suffer from poor crystallinity (the presence of defects and water molecules), resulting in that PBA-based metal ion charge carrier batteries often exhibit poor cycle life [70]. For most batteries, increasing crystallinity and reducing water content are the key strategy to improve the electrochemical performance of PBAs. It is thought-provoking that many previous studies have demonstrated that crystal water is of great significance to the transport of protons, so the structure design of electrodes for proton batteries should be quite different from conventional PBAs [76, 77]. Simultaneously, some studies have found that PBAs based on the Grotthuss mechanism had high proton conductivity due to the existence of a 3D hydrogen bond network formed between zeolite water [77, 78]. Wu et al. demonstrated that only CuFe-TBA with a continuous hydrogen bond network was the best host for proton storage, of which zeolite water and ligand water formed hydrogen bonds and connected them together to build a penetration hydrogen bond network when there were vacancies in adjacent sites [21]. The DFT calculation was used to track the migration of protons in the intercalation process: they first modeled protons combined with ligand water after entering, and then protons appeared in the position of the zeolite water after relaxation, indicating that zeolite waters were the best binding sites for protons (Fig. 5b–d). Besides, protons were transported through cooperative cleavage and reconstruction of the covalent and hydrogen bond, resulting in 0.15 eV activation energy, which indicated that the Grotthuss mechanism played a key role in the transport and storage of protons in CuFe-TBA (Fig. 5e). Finally, the CuFe-TBA electrode achieved super rate performance (the electrode delivered a specific capacity of 49 mAh g^−1^ at 4000 C) and unprecedented cycle life (capacity retention was 60% after 0.73 million cycles at 500 C) (Fig. 5f–g). Taking advantage of Grotthuss topochemistry will facilitate the effective combination of batteries and capacitors and provide new ideas for the development of high-energy and high-power electrochemical energy storage devices.

In addition, another Turnbull blue analog (NiFe-TBA) had also been reported, the NiFe-TBA cathode achieved an ultrahigh rate performance at 6000 C (390 A g^−1^) and a high capacity at − 40 °C [38]. Moreover, they used operando synchrotron XRD to evolve the proton reaction process, during charge and discharge only a slight shift occurred at a diffraction angle (200) (corresponding to the mutual conversion between [Fe^II^(CN)_6_].^4−^ and [Fe^III^(CN)_6_].^3−^). In situ X-ray absorption near-edge spectra (XANES) revealed that the valence state of Fe had changed, but the valence state of Ni had not changed in the whole process, which identified that Fe was the active center of the electrochemical reaction. Although PBAs have outstanding advantages in proton battery applications, low capacity is still a problem to be solved. On the other hand, V-based PBAs may be used as electrode materials for high-capacity proton batteries owing to the high theoretical capacity of V-based compounds and the multi-electron reaction of V elements [72]. Peng et al. reported a vanadium hexacyanoferrate (VHCF) with a high capacity of 108 mAh g^−1^ that was currently the highest capacity reported for PBA-based cathode electrode for proton batteries [75]. The high capacity of VHCF mainly came from two redox active centers of vanadium (V^III^ ↔ V^IV^ ↔ V^V^) and iron (Fe^II^ ↔ Fe^III^). Nevertheless, it was a pity that the VHCF did not display an outstanding rate and long cycle life compared with TBAs, the reason for which could be the lack of a continuous hydrogen bond network and the water solubility of vanadium-based PBAs. To summarize, there is still a lot of work to be done to increase the capacity of PBAs and reveal the transport and storage mechanism of protons.

#### Two-dimensional Transition Metal Carbides/Nitrides (MXenes)

**Fig. 6:**
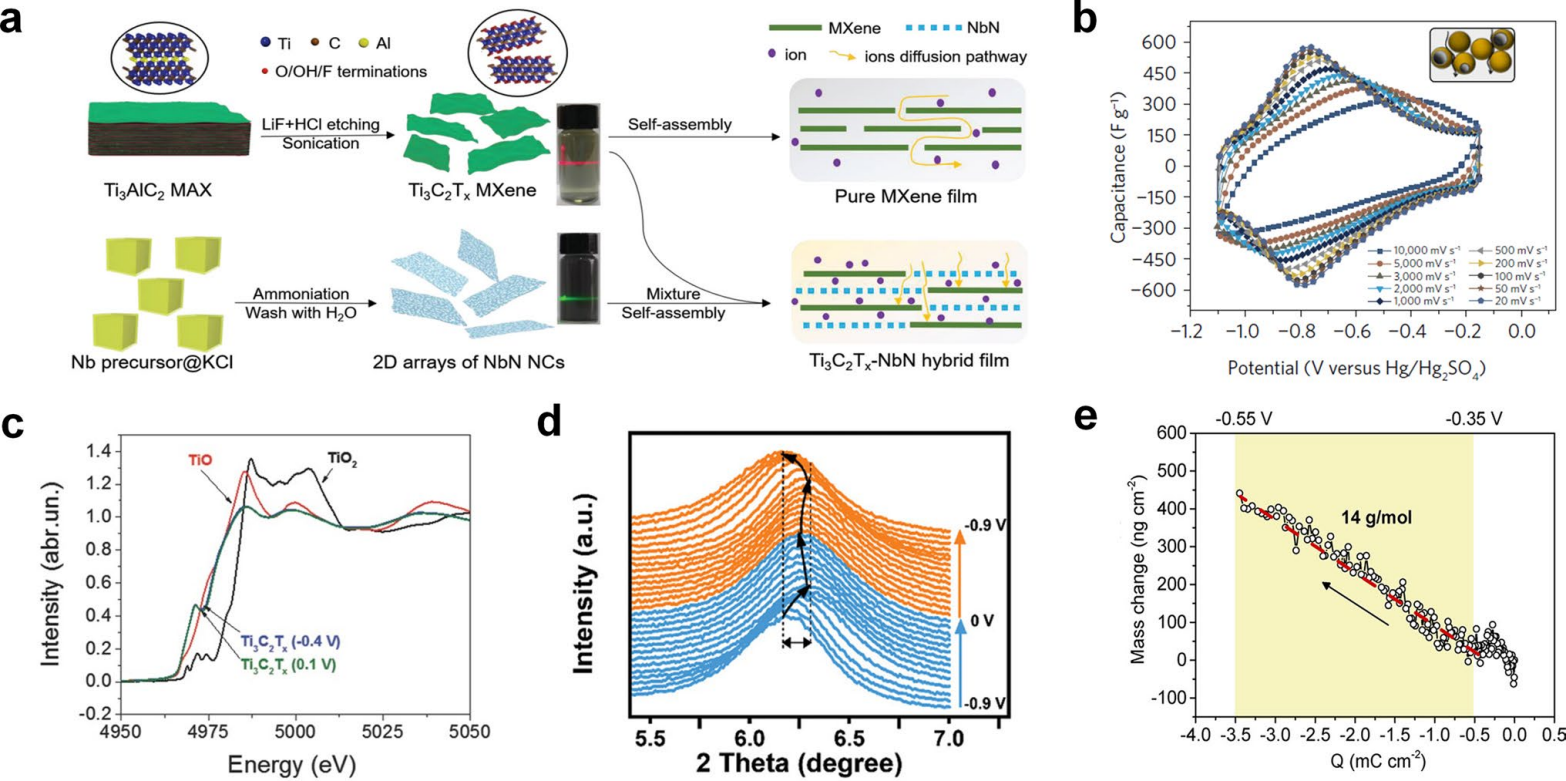
**a** Diagram of structure and synthesis of MXene nanosheets [118]. Copyright 2020, Wiley–VCH. **b** Capacitance of macroporous Ti_3_C_2_T_*x*_ electrode at various scan rates in 3 M H_2_SO_4_ [27]. Copyright 2017, Springer Nature. **c** Ti K-edge XANES spectra of Ti_3_C_2_T_x_ at − 0.4 and 0.1 V [83]. Copyright 2015, Wiley–VCH. **d** Electrochemical in situ X-ray diffraction study of Ti_3_C_2_T_*x*_ in 1 M H_2_SO_4_ [85]. Copyright 2019, Wiley–VCH. **e** MXene electrode mass and response charge changes in 3 M H_2_SO_4_ [86]. Copyright 2020, American Chemical Society

In 2011, an unprecedented 2D material MXene was reported [79]. Notably, MXenes are synthesized by HF acid etching MAX phase (*M*_*n* + 1_AX_*n*_, where *n* = 1, 2, 3, *M* is transition metal such as Ti and Mo, A is often group 13 or group 14 element, and X represents carbon or nitrogen) (Fig. 6a). Consequently, the unique structure of MXenes endows them with excellent electrochemical performance: On the one hand, the presence of internal metal carbides increases electrochemically active sites to realize the rapid transfer of electrons; on the other hand, the two-dimensional nanostructure and structural waters existing between the layers can promote superfast transport of ions. Besides, abundant surface functional groups produced during the synthesis process provide a large number of redox active sites for protons. Ti_3_C_2_T_*x*_, researched the most widespread among MXenes, has been comprehensively used in lithium-ion batteries [80], electric double-layer capacitors [81], pseudocapacitors [27], etc. In 2014, a method of preparing MXenes by etching MAX with a mixture of LiF and HF was reported by Ghidiu et al. (This reaction condition was milder than HF and beneficial to the decrease of nanosheet defects.) Additionally, a roller mill was used by them to mold a kind of high-conductivity ‘clay’ Ti_3_C_2_ film, which delivered high volumetric capacitance (900 F cm^−3^), excellent cycle life, and rate performance in 1 M H_2_SO_4_. Aiming to overcome the limitation of rate and voltage for MXenes, Lukatskaya et al. prepared a macroporous MXene coated on a glassy carbon current collector [27]. The presence of glassy carbon effectively extended the electrochemical window to a breakthrough potential (− 1.1 to − 0.1 V versus Hg/Hg_2_SO_4_) in the acidic electrolyte, the reason for which could be that the higher overpotential of glassy carbon was beneficial to reducing hydrogen evolution reaction (HER). Simultaneously, the macroporous structure of the film provided a channel for the rapid transport of protons and enhanced the kinetics of the electrode (the electrode delivered a specific capacitance of 210 F g^−1^ at 10 V s^−1^) (Fig. 6b). Furthermore, a 3 $\upmu$m Ti_3_C_2_T_*x*_ hydrogel electrode with a volumetric capacitance of 1500 F cm^−3^ that surpassed the volumetric capacity of all electrodes reported previously was found. In order to meet the actual demand for miniature devices and wearable electronic products, many MXene-based energy storage flexible devices have been developed. Unfortunately, inactive polymers (such as MMA) are often added into the electrode manufacturing process, resulting in a decrease in volumetric capacitance. Yu et al. took advantage of the excellent physical and chemical properties of antimonene (metal Sb nanosheets) to combine with MXene to form a uniform and dense high electroactive film, thereby improving the transport and storage of protons in MXene [82]. Finally, the specific capacity of the composite film had been greatly increased, and the volumetric capacitance value of 4255 F cm^−3^ was the largest value among the known MXene-based flexible electrodes.

Nevertheless, in sharp contrast to the vigorous development of MXene materials’ structure design, there is still a lack of in-depth understanding of the excellent performance of MXene electrodes in acid electrolytes. MXene exhibits nearly rectangular cyclic voltammetry (CV) curves in a sulfuric acid electrolyte. (This is similar to the electrochemical behavior of RuO_2_.) Lukatskaya et al. believed that such a high capacity was most likely to come from the intercalation pseudocapacitance rather than surface adsorption and desorption or surface redox [83]. To prove their hypothesis, they used X-ray absorption near edge structure spectroscopy (XANES) to detect the oxidation state changes of Ti elements at different potentials. The Ti edge shifted to lower energy as potential was negatively scanned from 0.275 to − 0.35 V, and it would shift to higher energy when the potential was back to 0.35 V (Fig. 6c). Therefore, the electrochemical reaction can be assumed as:

1 $${\text{Ti}}_{{3}} {\text{C}}_{{2}} {\text{O}}_{x} \left( {{\text{OH}}} \right)_{y} {\text{F}}_{z} + \delta {\text{H}}^{ + } + \delta {\text{e}}^{ - }\leftrightarrow {\text{Ti}}_{{3}} {\text{C}}_{{2}} {\text{O}}_{x - \delta } \left( {{\text{OH}}} \right)_{y + \delta } {\text{F}}_{z}$$

In the meantime, some studies had demonstrated that alkali metal cations in the electrolyte were inserted into Ti_3_C_2_T_*x*_ layers and then bound to the ends of the functional groups on the surface of MXenes [84]. Generally, it was believed that the intercalation and extraction of protons will not cause major structural changes because of the minimum ion radius of protons. However, the charge storage of MXene in sulfuric acid was much more complicated when obvious lattice expansion/shrinkage was found by in situ XRD during charge and discharge [85]. The lattice parameter of the *c*-axis shrunk by 0.1 Å during discharging from 0 to − 0.6 V and then expanded to 28.61 Å from 28.11 Å continued to discharge to − 0.9 V (Fig. 6d). More importantly, DFT simulations showed that the interlayer spacing of Ti_3_C_2_T_*x*_ with OH end groups expanded if proton inserted, while Ti_3_C_2_T_*x*_ with O end groups shrunk after proton intercalation, these results were consistent with in situ XRD results. To understand the role of water molecules in charge storage of Ti_3_C_2_T_*x*_, Shao et al. used a variety of in situ characterization techniques combined with molecular dynamics simulations to prove that hydronium ions instead of bare protons were inserted into electrodes during the electrochemical reaction (Fig. 6e) [86]. Besides, it was found that the presence of an appropriate amount of –OH would accelerate the reaction kinetics of Ti_3_C_2_T_*x*_ electrodes. (Many –OH groups would destroy the structure of water molecules and slow down the transport of ions, while few –OH groups would not be able to form a hydrogen bond network to trigger the Grotthuss mechanism.)

### Conversion Reaction Materials

#### ***MoO***_***3***_

**Fig. 7:**
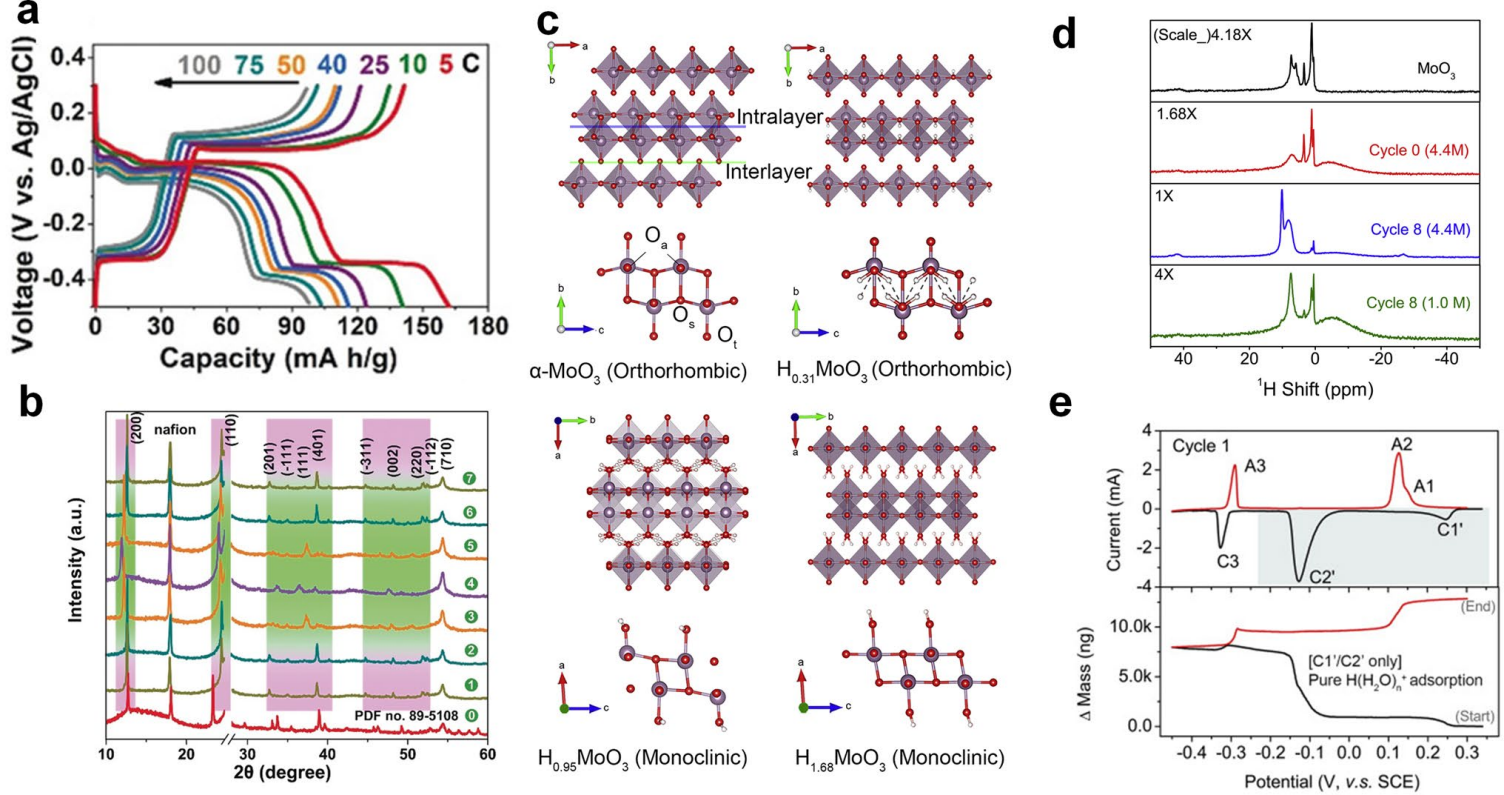
**a** Rate performance of MoO_3_ in 1 M H_2_SO_4_. **b** Ex situ XRD pattern of MoO_3_ at different potentials [28]. Copyright 2018, Wiley–VCH. **c** Structural changes of *α*-MoO_3_ during electrochemical reactions. **d** ^1^H Solid-state NMR of original MoO_3_ sample and cycled sample. **e** The CV and EQCM curves of MoO_3_ electrode in the first cycle [91]. Copyright 2020, Elsevier

MoO_3_ is a promising electrode material used in the battery field due to its extraordinary layered structure [87]. MoO_3_ is most widely studied in the orthorhombic phase, monoclinic phase, and hexagonal phase, among which orthogonal MoO_3_ (*α*-MoO_3_) is most favored by researchers because of its unique physical and chemical properties [88]. In 2018, an *α*-MoO_3_ electrode that delivered a discharge capacity of 152 mAh g^–1^ (5 C) in 1 M H_2_SO_4_ electrolyte was reported, and the capacity was 88 mAh g^−1^ at a rate of 100 C (Fig. 7a) [28]. Furthermore, the areal specific capacity reached 22.4 mAh cm^−2^ even under an ultra-high loading mass of 90 mg cm^−2^, and such excellent areal specific capacity was never reported in other metal ion charge carrier batteries [89]. A free-standing MoO_3_/Ti_3_C_2_T_z_ composite film electrode also achieved high gravimetric/volumetric capacities of 837 and 1836 C cm^−3^ owing to MoO_3_ nanobelts interconnected by MXene nanosheets [90]. However, it was puzzling that the redox peak in the CV curves was very sharp, unlike most electrochemical proton storage materials with a broad redox peak. Electrode kinetics studies showed that the kinetics of MoO_3_ was limited by the diffusion of hydrogen ions (*b* $\approx$ 0.5). Ex situ XRD was used to study the structural changes of the electrode during charging and discharging, a new phase H_0.84_MoO_3_ was generated with the emergence of some new peaks at 24.58°, 44.78°, and 48.28° from the initial potential to − 0.5 V (Fig. 7b). Then, new peaks appeared in pattern 3 and pattern 4 in the charging process, and the electrode returning to the initial potential was found to have a different structure from the initial structure (MoO_3_), revealing that the first charge and discharge process was irreversible. The subsequent electrochemical reaction was highly reversible, which was why MoO_3_ had a high-rate performance in acid electrolytes. The above reaction process was also confirmed by Yan et al. [40]. Nonetheless, although researchers have already understood the structural changes of MoO_3_ during charging and discharging, more subtle structural transformations require in situ characterization technique to identify. Guo et al. proved by in situ XRD that MoO_3_ underwent many complex solid-solution reactions and two-phase reactions during charge and discharge (Fig. 7c) [91]. First, the following reactions occurred during the first discharge:

2 $${\text{MoO}}_{{3}} \to {\text{H}}_{{0.{31}}} {\text{MoO}}_{{3}} \to {\text{H}}_{{0.{95}}} {\text{MoO}}_{{3}} \to {\text{H}}_{{{1}.{68}}} {\text{MoO}}_{{3}}$$

Then, the reaction process during the first charging process followed the following equation:

3 $${\text{H}}_{{{1}.{68}}} {\text{MoO}}_{{3}} \to {\text{H}}_{{0.{95}}} {\text{MoO}}_{{3}} \to {\text{H}}_{{0.{5}}} {\text{MoO}}_{{3}}$$

Finally, the following reversible electrochemical reaction occurred in the next cycles:

4 $${\text{H}}_{{0.{5}}} {\text{MoO}}_{{3}} \leftrightarrow {\text{H}}_{{{1}.{68}}} {\text{MoO}}_{{3}} \leftrightarrow {\text{H}}_{{0.{95}}} {\text{MoO}}_{{3}} \leftrightarrow {\text{H}}_{{0.{5}}} {\text{MoO}}_{{3}}$$

The above evidence also suggested that only intercalation/deintercalation of pure protons instead of water molecules or hydronium ions appeared in MoO_3_ and hydrogen molybdenum bronzes (HMBs) during charging and discharging. What kind of role did water molecules play? Solid-state NMRs provided us with some information that hydronium ions only existed on the surface instead of the bulk lattice of the electrode (Fig. 7d). Simultaneously, the change of electrode mass that was detected by EQCM during the first two stages was caused by the adsorption of hydronium ions; then, water molecules stayed on the surface of the electrode and protons were inserted into the electrode material (Fig. 7e). During the charging process, protons were extracted from the bulk of the electrode with the opposite transport of water, causing a further increase in the mass of the electrode. As the content of absorbed and desorbed water molecules tended to be balanced, the charge transfer of the electrode was enhanced, and the electrode kinetics had been greatly improved by the synergistic effect of protons and water molecules.

#### Organic Materials

**Fig. 8:**
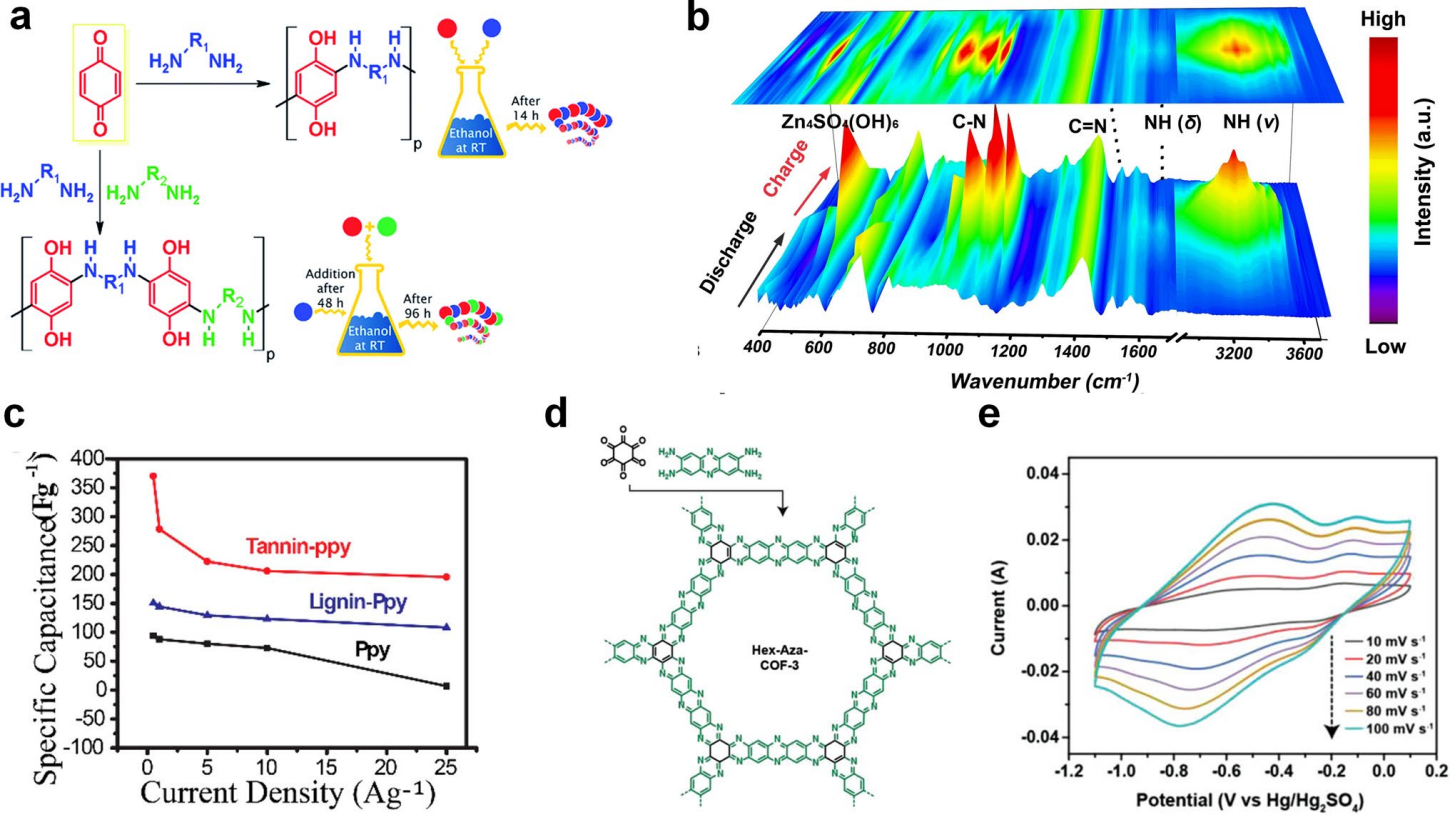
**a** Schematic of preparation of bipolymer and terpolymer [94]. Copyright 2017, The Royal Society of Chemsitry. **b** Ex situ FTIR spectra of PO electrodes during charging and discharging process [96]. Copyright 2021, Wiley–VCH. **c** Specific capacitance of Tn/Ppy, Lig/Ppy, and Ppy at different current densities [97]. Copyright 2017, American Chemical Society. **d** The synthetic route of Hex-Aza-COF-3. **e** CV curves of Hex-Aza-COF-3 at various scan rates [100]. Copyright 2020, Wiley–VCH

Organic materials, a type of conversion electrode materials, have been widely used in the field of energy storage for their low cost and diverse designability. Actually, the active sites of organic compounds can reversibly combine with protons in the electrolyte so that organic electrode materials can deliver higher specific capacity, but there are some problems such as poor conductivity and low stability [92]. A class of materials containing carbonyl functional groups (C=O) in organic compounds is used for electrochemical proton storage, such as benzoquinones and ketones [33, 93–95]. Two new redox-active bis- and terpolymers containing quinone-amine were synthesized by Navarro-Suarez et al., and the reaction between diamine and benzoquinone significantly enhanced the redox activity of polymers due to the generation of more active centers that were conducive to storaging more protons. Therefore, the poly(benzoquinone-co-hexamethylenediamine-co-PEO/PPO) electrode delivered a specific capacity of 230 mAh g^−1^ at a current density of 0.08 A g^−1^ (Fig. 8a) [94]. Moreover, it was revealed that the formation of intramolecular hydrogen bonds contributed to the transfer of protons and electrons via Fourier transform infrared spectroscopy (FTIR), facilitating the electrochemical reaction. Guo et al. also developed an organic pyrene-4,5,9,10-tetraone (PTO) that delivered a specific capacity of 208 mAh g^−1^ at 0.16 mA cm^−2^ as well as an outstanding rate performance of 85 mAh g^−1^ at 480 mA cm^−2^ [33]. Other organic electrode materials with C=N groups or N=N groups can also achieve reversible proton storage. A poly(2,9-dihydroquinoxalino[2,3-*b*] phenazine) (PO) organic material was synthesized to design an all-organic proton battery that delivered a specific capacity of 147 mAh g^−1^ with a long-term cycle life of over 500 cycles in mild electrolyte [96]. The chemical environments of adjacent C=N groups in PO molecules are different enough to result in a two-step redox reaction (Fig. 8b), PO molecules achieved reversible H^+^ uptake/removal behavior in ZnSO_4_ electrolyte:

5 $${\text{2H}}_{{2}} {\text{O}} \leftrightarrow {\text{2n H}}^{ + } + {\text{2n OH}}^{ - }$$

6 $${\text{PO}} + {\text{2n H}}^{ + } + {\text{2n e}}^{ - } \leftrightarrow {\text{P}}$$

7 $$\begin{aligned}& {\text{n Zn}}^{{{2} + }} + {\text{2n OH}}^{ - } + {\text{n}}/{\text{3 ZnSO}}_4 + {\text{5n}}/{\text{3 H}}_{{2}} {\text{O}} \\ &\quad \leftrightarrow {\text{ n}}/{\text{3 Zn}}_{{4}} {\text{SO}}_{{4}} \cdot {\text{OH}}_{{6}} \cdot{\text{5H}}_{{2}} {\text{O}} \\ \end{aligned}$$

Besides, many conductive polymers (CPs) [97–99] and covalent organic frameworks (COFs) [100, 101] have been developed as electrochemical proton storage materials. Conductive polymers with poor stability are often compounded with other substances to improve electrochemical performance. Mukhopadhyay et al. used environmentally friendly solvents to extract tannic acid with high phenol content (5.56 mol g^−1^) from the bark [97]. The composite electrode of tannic acid and conductive polypyrrole (Ppy) achieved the highest specific capacitance of 370 F g^−1^ at a current density of 0.5 A g^−1^, even the specific capacity remained 196 F g^−1^ at a high current density of 25 A g^−1^ (Fig. 8c). Likewise, a polypyrrole (PPy)/2D Cu-TCPP (TCPP = 5,10,15,20-tetrakis(4-carboxyphenyl)porphyrin) composite electrode with a compact composite network structure also achieved a specific capacitance of ∼500 F g^−1^ in 0.5 M H_2_SO_4_ [98]. Covalent organic frameworks (COFs), a new type of crystalline organic porous materials, possess excellent structure, which endows them with great application potential in the fields of gas separation, photovoltaics, energy storage, catalysis, etc. [102]. Yang et al. designed two COFs with high crystallinity, stability, and porosity (NKCOF-2 and NKCOF-8), and the CNT/NKCOF-2 composite electrode exhibited a specific capacity of 440 F g^−1^ at 0.5 A g^−1^. FT-IR, PXRD, Raman spectroscopy, and EIS revealed that protons were transferred to active site (azo groups) via the Grotthuss conduction mechanism to achieve reversible conversion reactions (N=N + 2H^+^  + 2e^−^  ↔ H–N–N–H). Kandambeth et al. reported two kinds of COFs (Hex-Aza-COF-2 and Hex-Aza-COF-3), which were synthesized by cyclohexanehexone and redox-functionalized aromatic tetramer with benzoquinone or phenazine in solvothermal condensation reaction (Fig. 8d) [100]. Unexpectedly, the electrochemical window of these two COFs electrodes in 1 M H_2_SO_4_ reached − 1 V (vs Hg/Hg_2_SO_4_), and the specific capacitance of Hex-Aza-COF-3 even achieved 663 F g^−1^, which exceeded all reported COFs materials (Fig. 8e). The asymmetric device (RuO_2_//Hex-Aza-COF-3) with a wide voltage of 1.7 V delivered an energy density of 23.3 Wh kg^−1^ at a power density of 661.2 W kg^−1^. At present, the research on organic electrode materials for electrochemical proton storage is still in the preliminary stage, and further research on the synthesis, characterization, and charge storage mechanism of organic electrodes needs to be further explored. Lots of reported electrode materials for electrochemical proton storage are summarized in Table 1.

**Table 1:** Summary of electrode materials for electrochemical proton storage

Material	Electrolyte	Potential (V)	Capacity (mAh g^–1^)	Rate (mAh g^–1^)	Cycle life (no. of cycles)	References
RuO_2_·*x*H_2_O	0.5 M H_2_SO_4_	0.2 to 1.2	417 (20 A g^−1^)	319 (150 A g^−1^)	–	[119]
RuO_2_/Graphite	1 M H_2_SO_4_	0.2 to 1.2	173 (0.1 A g^−1^)	126 (20 A g^−1^)	5000 (80%)	[120]
WO_3_·0.6H_2_O	0.5 M H_2_SO_4_	− 0.4 to 0.5	124 (5 mV s^−1^)	99 (100 mV s^−1^)	50,000 (100%)	[26]
H_2_W_2_O_7_	3 M H_2_SO_4_	− 0.2 to 0.8	70 (1 mV s^−1^)	56 (1 V s^−1^)	100,000 (89%)	[121]
CuFe-TBA	2 M H_2_SO_4_	0 to 1.3	95 (9.5 mA g^−1^)	49 (380 A g^−1^)	0.73 million (60%)	[21]
NiFe-TBA	1 M H_2_SO_4_	0 to 1.2	65 (0.1 A g^−1^)	39 (390 A g^−1^)	1000 (73%)	[38]
VHCF	6 M H_2_SO_4_	0.24 to 1.44	108 (0.1 A g^−1^)	65 (10 A g^−1^)	25,000 (92%)	[75]
Ti_3_C_2_T_*x*_	3 M H_2_SO_4_	−0.45 to 0.55	125 (10 mV s^−1^)	58 (10 V s^−1^)	10,000 (90%)	[27]
*n*-F-Ti_3_C_2_T_*x*_	3 M H_2_SO_4_	−0.2 to 0.4	87 (2 mV s^−1^)	–	10,000 (96%)	[122]
S-etched Ti_3_C_2_T_*x*_	3 M H_2_SO_4_	−0.55 to 0.55	91 (5 mV s^−1^)	58 (10 V s^−1^)	10,000 (99%)	[123]
MoO_3_	1 M H_2_SO_4_	−0.3 to 0.5	152 (0.2 A g^−1^)	88 (2 A g^−1^)	100 (67%)	[28]
MoO_3_	6 M H_2_SO_4_	−0.26 to 0.54	235 (1 A g^−1^)	174 (40 A g^−1^)	5000 (87%)	[89]
Tn/Ppy	0.5 M H_2_SO_4_	0.3 to 0.9	62 (0.5 A g^−1^)	33 (25 A g^−1^)	~ 9000 (~ 100%)	[97]
PTO	2 M MnSO_4_/H_2_SO_4_	0.2 to 0.9	208 (0.16 mA cm^−2^)	85 (400 mA cm^−2^)	1000 (67%)	[33]
Hex-Aza-COF-3	1 M H_2_SO_4_	−0.45 to 0.75	221 (1 A g^−1^)	–	–	[100]
CNT/NKCOF-2	2 M H_2_SO_4_	0 to 1.2	147 (0.5 A g^−1^)	72 (20 A g^−1^)	10,000 (91%)	[124]
NPenes	3 M HCl	0.2 to 1.2	395 (2 mV s^−1^)	–	–	[30]
2-GCE	2 M H_3_PO_4_	−0.5 to 0.65	284 (15 A g^−1^)	57 (15 A g^−1^)	–	[125]
W_2_N	1 M H_2_SO_4_	−0.4 to 0.8	159 (2 mA cm^−2^)	99 (20 mA cm^−2^)	10,000 (92%)	[29]

All potentials are referenced to a standard hydrogen electrode (where Ag/AgCl *vs.* SHE = 0.2 V, Hg/Hg_2_ Cl_2_ *vs.* SHE = 0.24 V, and  Hg/Hg_2_SO_4_ *vs.* SHE = 0.65 V). Specific capacitance is converted to specific capacity by the following equation: *C* = $\frac{3.6Q}{\mathrm{V}}$

## Device Configuration 

**Fig. 9:**
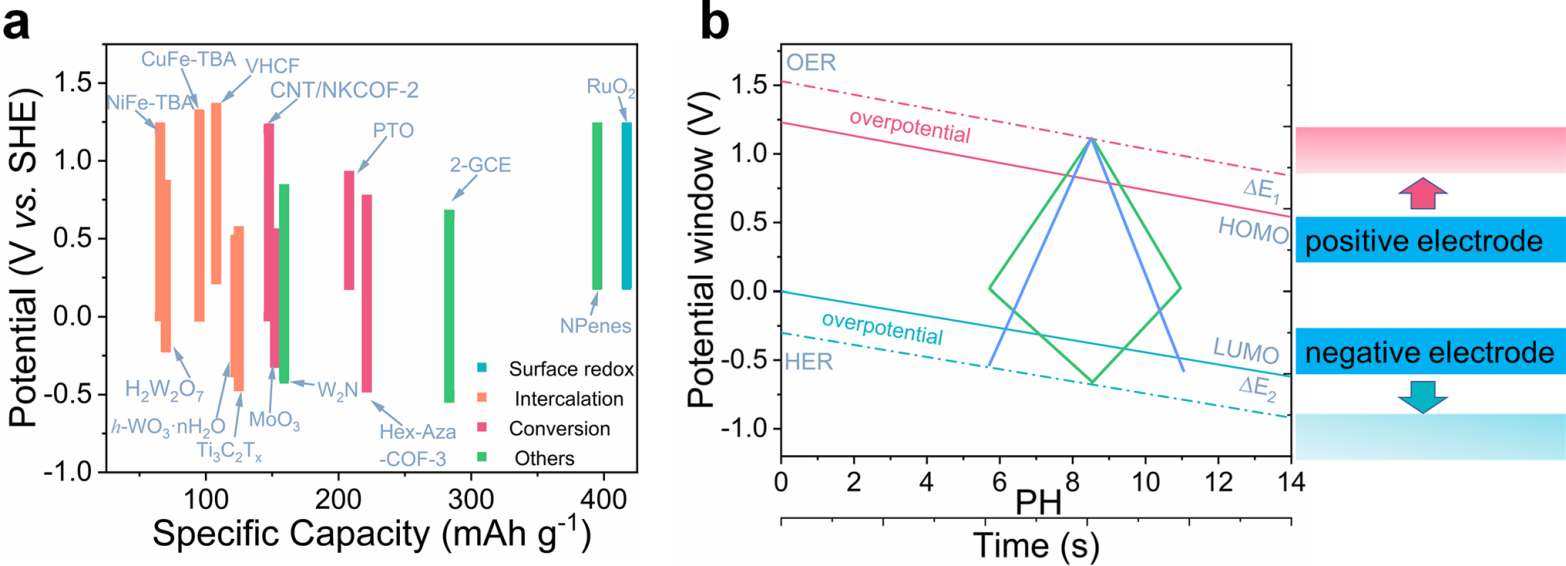
**a** Summary of the potential window of various electrode materials for EPS. **b** Schematic of potential window of a water-based full cell at different PH

**Fig. 10:**
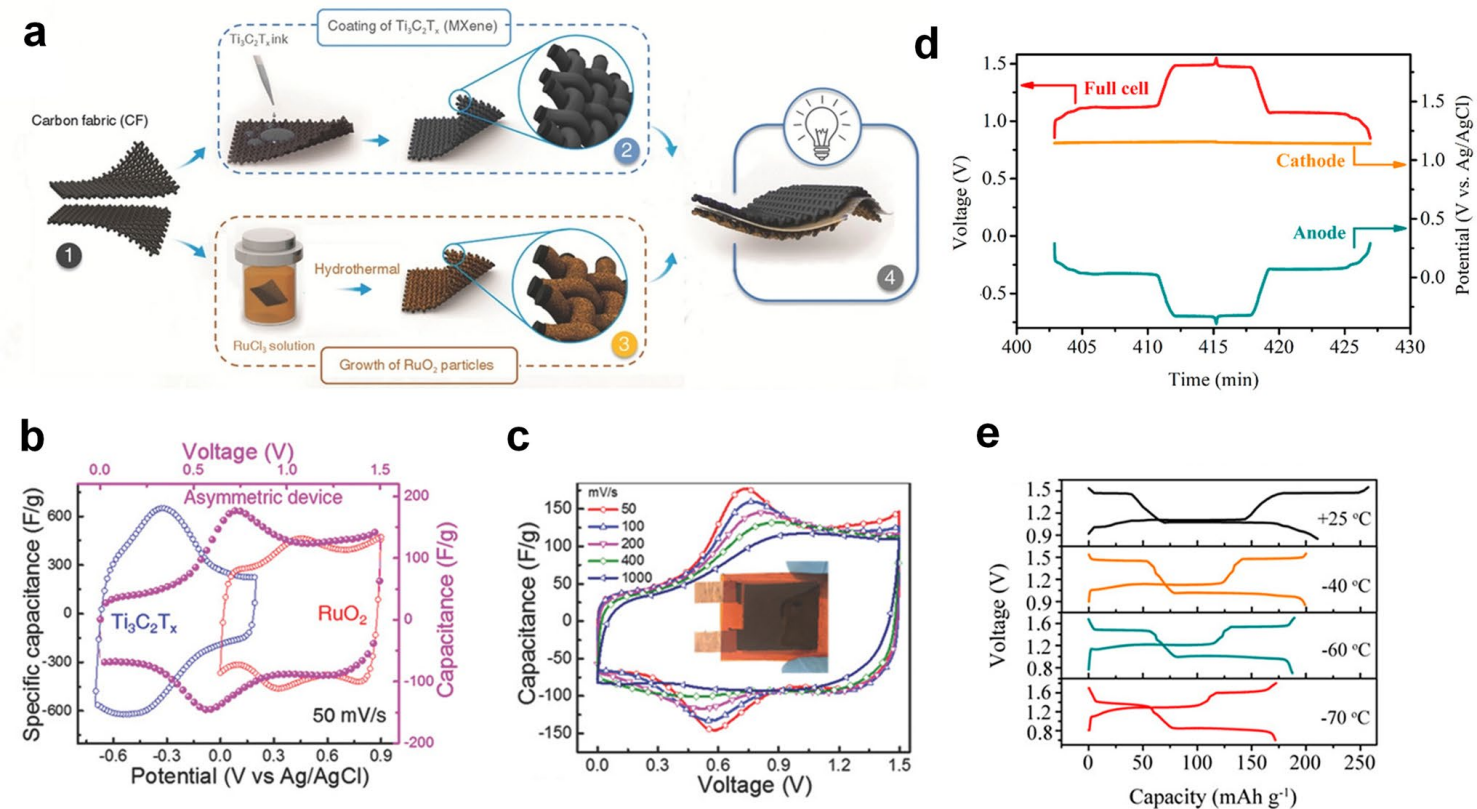
**a** The fabrication process of RuO_2_//Ti_3_C_2_T_*x*_ asymmetric proton pseudocapacitor. **b** In situ tracked variation of the potential in RuO_2_/CF electrode, Ti_3_C_2_T_*x*_/CF electrode, and RuO_2_//Ti_3_C_2_T_*x*_ asymmetric device. **c** Capacitance of RuO_2_//Ti_3_C_2_T_*x*_ asymmetric device at different scan rates [103]. Copyright 2018, Wiley–VCH. **d** The comparison of galvanostatic charge–discharge curves of MnO_2_@GF//MoO_3_ full cell, MnO_2_@GF cathode, and MoO_3_ anode at 1 mA cm^−2^. **e** Galvanostatic charge–discharge curves of MnO_2_@GF//MoO_3_ full cell at different temperatures [40]. Copyright 2020, American Chemical Society

The goal of the research on materials and charge storage mechanisms of electrochemical proton storage is to develop more efficient batteries/capacitors and lead the way to its industrialization. To achieve the above goals, it is very necessary to build a complete full cell device (Fig. 9a–b). Jiang et al. designed an all pseudocapacitive asymmetric supercapacitor using MXene (Ti_3_C_2_T_*x*_) as the negative electrode and ruthenium oxide (RuO_2_) as the positive electrode (Fig. 10a) [103]. This asymmetric supercapacitor operated at a voltage of 1.5 V in 1 M H_2_SO_4_, which far surpassed other MXene-based supercapacitors (Fig. 10b). More importantly, this supercapacitor exhibited extraordinary rate performance with a capacity retention of 84% at a high scan rate of 1000 mV s^−1^ (relative to the specific capacitance at 50 mV s^−1^), resulting in that the asymmetric device delivered an energy density of 24 Wh kg^−1^ at a power density of 26 kW kg^−1^ (Fig. 10c). Boota et al. demonstrated some working asymmetric proton pseudocapacitors using MXene (Ti_3_C_2_T_*x*_) as the negative electrode and conducting polymers (PANI@rGO, PPy@rGO, and PEDOT@rGO) as the positive electrode [104]. Owing to the complementary electrochemical window of positive and negative materials and the pseudocapacitive reaction derived from protons, the voltage window of these all pseudocapacitors reached 1.45 V in 3 M H_2_SO_4_. Moreover, the presence of rGO not only increased the conductivity of CPs but also improved the cycle stability of electrodes, and the asymmetric device of PANI@rGO//Ti_3_C_2_T_*x*_ showed capacitance retention of ≈ 88% after 20,000 cycles. Therefore, the composite electrode is a strategy that can be considered in the construction of a full cell, especially in improving the cycle stability of a device. An organic–inorganic all-pseudocapacitive supercapacitor with a MXene anode and a 2,5-dihydroxy-1,4-benzoquinone (DBQ)@rGO cathode was also investigated by Boota et al. [105]. Recently, our group reported an all proton pseudocapacitor with a Prussian analog (Cu_0.82_Co_0.18_HCF) as cathode and WO_3_·nH_2_O as anode with a voltage of 1.7 V, which delivered an energy density of 22 Wh kg^−1^ at a high power density of 26 kW kg^−1^ [106].

Jiang et al. designed a full proton battery using H-TBA as cathode, MoO_3_ as the anode, and 9.5 M H_3_PO_4_ as the electrolyte, which delivered a high capacity of 46 mAh g^−1^ at 5 A g^−1^ and capacity retention of 70% at100 A g^−1^. 9.5 M H_3_PO_4_ with a freezing point of below −88 °C enabled this full proton battery to exhibit a specific capacity of 28 mAh g^−1^ and specific energy density of 24 Wh kg^−1^ at an ultra-low temperature of −78 ℃. With the discovery of the co-intercalation mechanism of protons and metal ions in many materials (such as MnO_2_ and V_2_O_5_) [107, 108], some multi-ion batteries have been developed. Yan et al. developed a full cell using MoO_3_ as the anode and MnO_2_ as the cathode in a mixed electrolyte (2 M H_2_SO_4_ + 2 M MnSO_4_) (Fig. 10d) [40]. Therefore, the following reactions occurred in cathode and anode:

8 $${\text{cathode}}{:}\quad {\text{ Mn}}^{{{2} + }} + {\text{ 6H}}_{{2}} {\text{O }} \to {\text{ MnO}}_{{2}} \downarrow + {\text{ 4H}}_{{3}} {\text{O}}^{ + } + {\text{ 2e}}^{ - }$$

9 $${\text{anode}}{:}\quad {\text{ xH}}_{{3}} {\text{O}}^{ + } + {\text{ xe}}^{ - } + {\text{ MoO}}_{{3}} \to \, \left( {{\text{H}}_{{3}} {\text{O}}} \right)_{{\text{x}}} {\text{MoO}}_{{3}}$$

Then, the MnO_2_@GF//MoO_3_ full cell with a 1.6 V voltage window achieved a maximum energy density of 177.4 Wh kg^−1^ at a power density of 895.9 W kg^−1^, which was the highest energy density reported to date for a proton full cell (Fig. 10d). In particular, the acid solution with high conductivity not only increased the ioni c conductivity of the mixed electrolyte but also significantly reduced the freezing point of the mixed electrolyte. Finally, this full cell still possessed a specific capacity of 171.8 mAh g^−1^ at an ultralow low temperature of – 70 °C and cycled for 100 cycles without capacity fading (Fig. 10e). A similar full cell with a MnO_2_@graphite felt cathode and a pyrene-4,5,9,10-tetraone (PTO) anode was also reported by Guo et al. [33]. Similarly, this organic/inorganic full cell had excellent electrochemical performance (energy density of 132.6 Wh kg^−1^) and low-temperature performance (nearly 72% of the room-temperature capacity at −70 °C). This strategy of proton–metal ion hybrid full cell efficiently takes advantage of the high energy of metal ion batteries and high power of proton batteries and provides a new avenue for the construction of proton full cell. A very novel proton–gas battery with an H_2_ anode electrode, CuHCF cathode electrode, and 9 M H_3_PO_4_ electrolyte was also developed, which delivered a long cycle life over 0.35 million cycles [109]. More importantly, this CuHCF–H_2_ cell achieved an outstanding rate capacity of 30 mAh g^−1^ and cycled steadily over 1150 cycles at −60 °C. Some manganese–hydrogen batteries and nickel–hydrogen batteries with high energy, long life, and low cost have been successfully produced commercially for large-scale energy storage. Proton electrochemical energy storage devices not only achieve high energy density and power density but also show outstanding application value at extremely low temperatures [110, 111].

## Summary and Outlook

The development of energy internet and energy revolution technology is pushing for the application of electrochemical energy storage (EES) in various industries, and EPS will occupy an important place in the development of energy. Compared with traditional metal ion charge carrier batteries or capacitors, proton batteries and pseudocapacitors have been extensively studied for combined advantages of high capacity, superior rate, and long life in recent years. Specifically, this review systematically summarizes and discusses the preparation technology, electrochemical performance, and charge storage mechanism for the existing proton battery-type/ pseudocapacitive materials. According to different charge storage mechanisms, it is mainly classified into surface redox type, intercalation type, and conversion type. Although some progress has been made in proton electrochemical energy storage, it is still a key challenge to develop electrode materials for electrochemical proton storage with excellent performance, low cost, and high safety. Then, we also summarized the progress of the construction of EPS devices in detail, which enlightened significance for our understanding of this new type of energy storage system. Therefore, there is still more work that needs to be done to thoroughly understand the basic mechanisms of electrochemical proton storage and to build superior energy storage systems.

### Mechanism Understanding of EPS

The solid-phase reaction stage of common lithium-ion batteries is a typical diffusion-controlled process with a time scale of a few minutes, which makes it difficult to increase the power density of the lithium-ion batteries. In recent years, the vigorous development of the energy storage industry has increased the demand for power batteries, increasing people's interest in exploring electrode materials with high diffusion coefficients. Although proton battery-type/pseudocapacitive materials undergo an intercalation/conversion reaction similar to the battery during charge and discharge, most of them are nondiffusion-controlled on the time scale, so most of them can be considered as pseudocapacitive in nature. Furthermore, proton battery-type/pseudocapacitive materials seem to be a potential opportunity that achieves high power density and energy density because it combines the advantages of batteries and capacitors. However, there is still unclear as to why protons are transported so rapidly in these electrode materials. Though some people thought that protons could be rapidly transported on the hydrogen bond network through the Grotthuss diffusion mechanism, there was no direct evidence so far. The key parameters such as the physical and chemical properties of protons, interface effects, and energy changes will be effectively combined through theoretical calculations, and using cutting-edge theory establishes a reasonable structural model from the atomic scale to understand the charge storage mechanisms of EPS. Advanced in situ characterization techniques are also indispensable for tracing transport pathways of protons and revealing binding sites of protons and electrode materials during proton storage. The determination of the interaction between electrode materials and protons will be of significance for the design of advanced proton battery-type/pseudocapacitive materials.

### Electrode Design of EPS

Electrode materials are of paramount importance to improving the electrochemical performance of EPS. The anode material with a higher overpotential that is conducive to reducing hydrogen evolution reaction (HER) is a suitable host for proton storage [112]; current anode materials are concentrated on W-based, Mo-based, and Ti-based electrode materials in the light of Trassati’s volcano plot [113]. More anode materials with high-capacity, high-rate performance, and cyclic stability can be developed through designing rational lattice structures and regulating structural waters to build a continuous hydrogen bond network. Additionally, greater efforts are needed to design efficient high-voltage-resistant cathode materials because there is that only Prussian blue analogue cathode materials deliver good electrochemical performance. Polyanionic compounds (such as VOPO_4_) with high theoretical capacity and theoretical redox potential have been designed as electrode materials for lithium-ion batteries, which are also promising high-voltage cathode materials for EPS. On the one hand, it is important to understand the influence of defect structure on electrochemical performance, which may help us optimize synthesis methods to add more active sites to store more protons. On the other hand, water molecules (including free water and structural water) and surface functional groups in electrode materials have a significant impact on their electrochemical performance. Reasonable defect distribution and appropriate water content/surface groups will facilitate the building of a complete hydrogen bond network, which may be a highway for rapid proton transport. Therefore, it is crucial to explore safe, reliable, and low-cost routes to prepare more controlled electrode materials with high capacity and wide potential (especially high-voltage cathode materials).

### Device Configuration of EPS 

The current application research on EPS is mainly concentrated in acidic aqueous solution, so that proton batteries/pseudocapacitors deliver a low energy density owing to the decomposition of water under a lower voltage. The research focus of EPS can be appropriately shifted to the development of electrolytes with a wide electrochemical window, such as aqueous hybrid electrolytes (additive, water-organic, etc.), organic acids, and proton ionic liquids for their large decomposition voltage windows [114]. Recently, some work on widening the electrochemical windows of aqueous electrolytes has been reported [115, 116]. For example, adding some sodium dodecyl sulfate (SDS) electrolyte to water led the voltage window to expand to 2.5 V because of the reason that the hydrophobic layer formed by SDS electrostatically adsorbed to the electrode surface and effectively prevented the generation of OER/HER [116]. How to optimize aqueous electrolytes preparation or regulate high-efficiency organic protonic electrolytes and explore the mechanism and dynamics of electrochemical proton storage are awaiting further research. Moreover, another effective way to increase operating voltage is to build full-cell configurations and maintain a good charge balance, which can make full use of the positive and negative voltage windows, which has been confirmed to be the easiest way to expand the operating voltage of electronic double-layer capacitors and pseudocapacitors [117]. It is also a good way to develop proton-metal ion hybrid batteries to maximize energy and power density by combining the rapid kinetics of protons with the high energy of metal ions. More matching strategies of positive and negative electrodes need to be further explored, which is i mportant to the development of electrochemical proton storage devices with high specific energy and power.

### Practical Application of EPS

To meet the demand for grid-scale energy storage, the practical application of EPS devices must be developed. Proton batteries and pseudocapacitors have advantages of simultaneously maximizing energy density and power density, to bridge the gap between metal-ion batteries and capacitors. Proton batteries/capacitors will occupy some special markets in the future. The fast kinetics of proton electrochemical energy storage not only endow proton batteries/capacitors with higher rate characteristics compared to metal-ion batteries, but also the potential to operate at ultra-low temperatures. On the one hand, freezing points of electrolytes generally will be lowered by using acidic electrolytes rich in protons (such as “water-in-acid” electrolytes). On the other hand, protons can be transported inside the lattice via the Grotthuss mechanism (over 60% capacity retention below –60 ℃ for many proton batteries/capacitors), while the transport speed of metal ions in liquid and solid states decreases significantly with decreasing temperature (~ 10% capacity retention at –40 ℃ for LIBs). Further electrolyte optimization and electrode material structure design will facilitate the practical application of EPS in harsh environments such as polar, aerospace, and military weapons. Besides, the development of the connected and smart environment will promote the applications of EPS in such an emerging application field; therefore, it is very necessary to build compact, efficient, and miniaturized micro-scale energy storage devices for EPS [19]. To meet the needs of flexible and wearable technology, the structural design of proton batteries and pseudocapacitors must be combined with advanced technologies such as laser-based techniques and 3D printing to build better proton micro-batteries/micro-supercapacitors.
